# Supplementary material for: The shape of watersheds
Source: Nat Commun. 2018 Sep 17;9:3791. doi: 10.1038/s41467-018-06210-4 (PMC6141598; doi:10.1038/s41467-018-06210-4)
Supplement: Supplementary file 1 — Supplementary Information [file 41467_2018_6210_MOESM1_ESM.pdf]

The shape of watersheds

Sassolas-Serrayet et al.

List of authors:

Timothée Sassolas-Serrayet[1]*, Rodolphe Cattin[1], Matthieu Ferry[1]
[1] Géosciences Montpellier, Université de Montpellier and CNRS UMR 5243, 34090 Montpellier, France.* e-mail: timothee.sassolas-serrayet@umontpellier.f

Supplementary Information

Contents
Supplementary Figure 1 to 7

Introduction
This supporting information contains inversion results for n and c associated with basins in Bhutan, Southeast Africa, Iowa, Coastal Oregon and Pyrenees ; the location of elongated and compact basins in Bhutan Himalaya ; and the effect of DEM resolution.

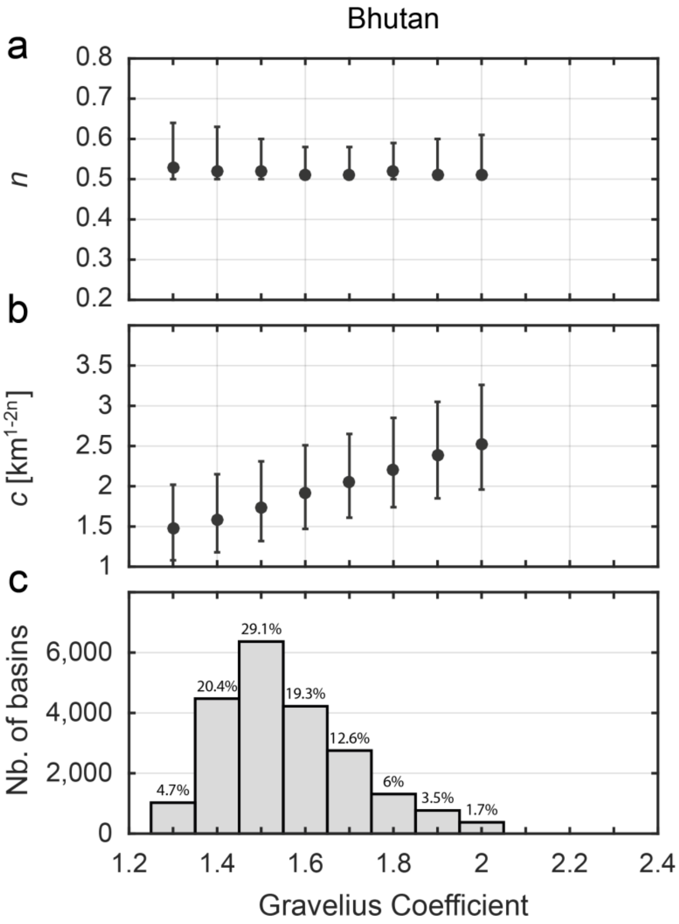

Supplementary Figure 1 | Hack's Law parameters vs. Gravelius coefficient in Bhutan Himalaya. Exponent and coefficient for each class of GC. Error bars are defined considering 95% of likelihood**. c,** Number of basins for each class of GC and its associated proportion.

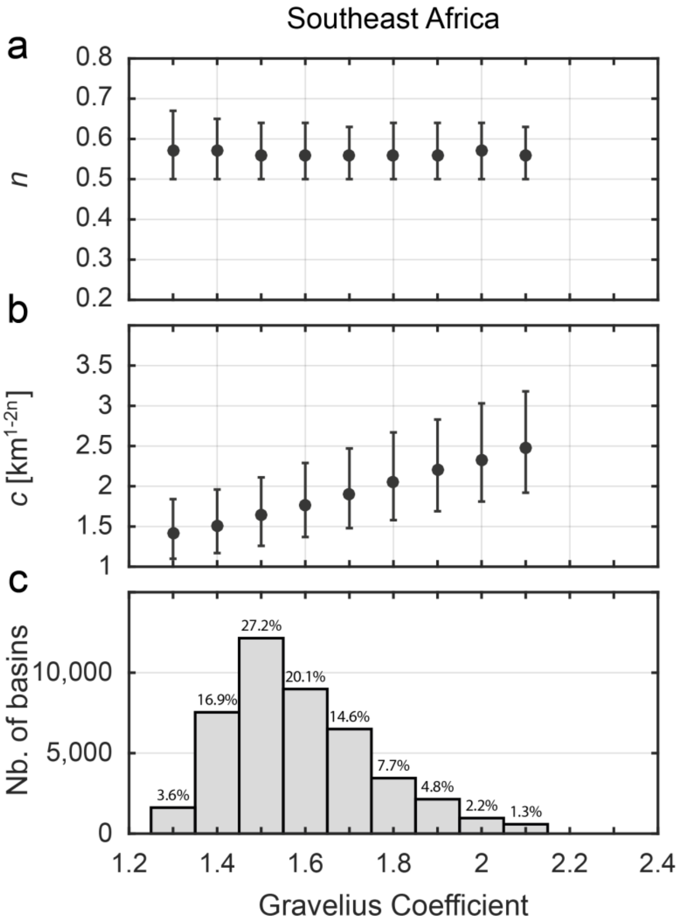

Supplementary Figure 2 | Hack's Law parameters vs. Gravelius coefficient in south-east Africa. Same as supplementary Figure 2, for south-east Africa.

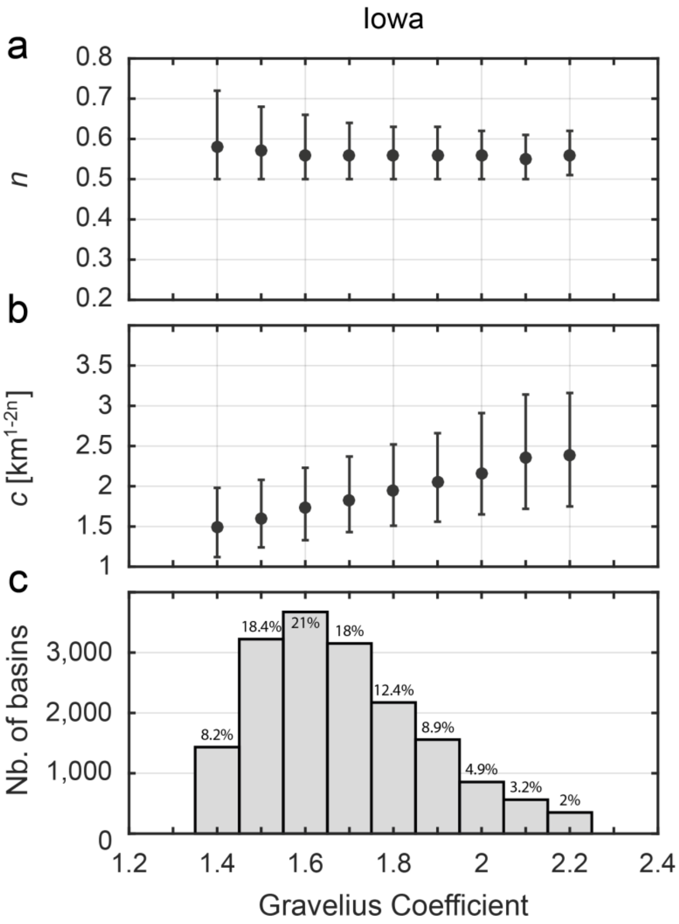

Supplementary Figure 3 | Hack's Law parameters vs. Gravelius coefficient in Central Iowa. Same as supplementary Figure 2, for Central Iowa.

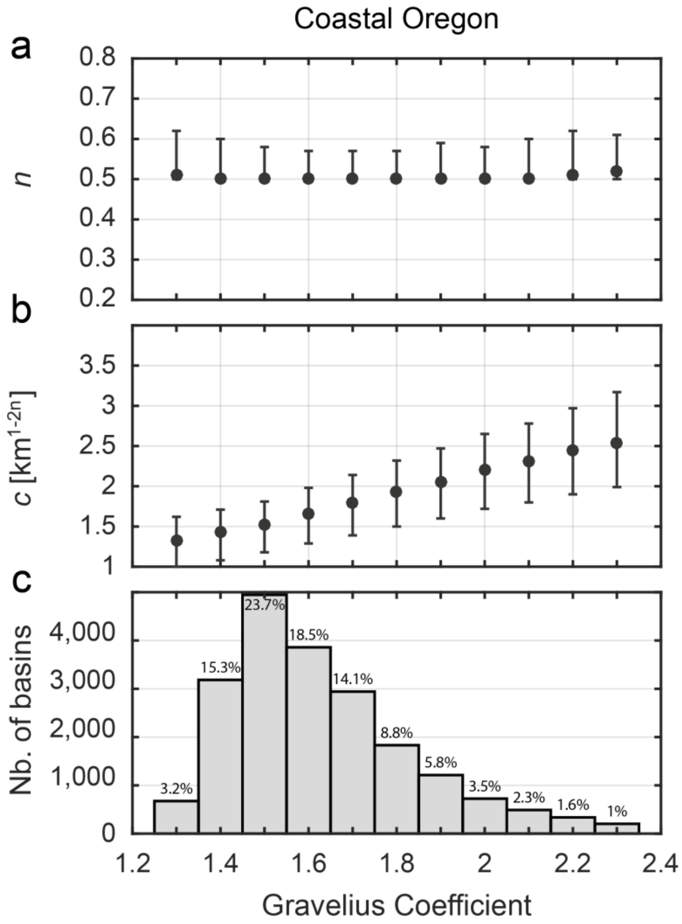

Supplementary Figure 4 | Hack's Law parameters vs. Gravelius coefficient in Coastal Oregon. Same as supplementary Figure 2, for Coastal Oregon.

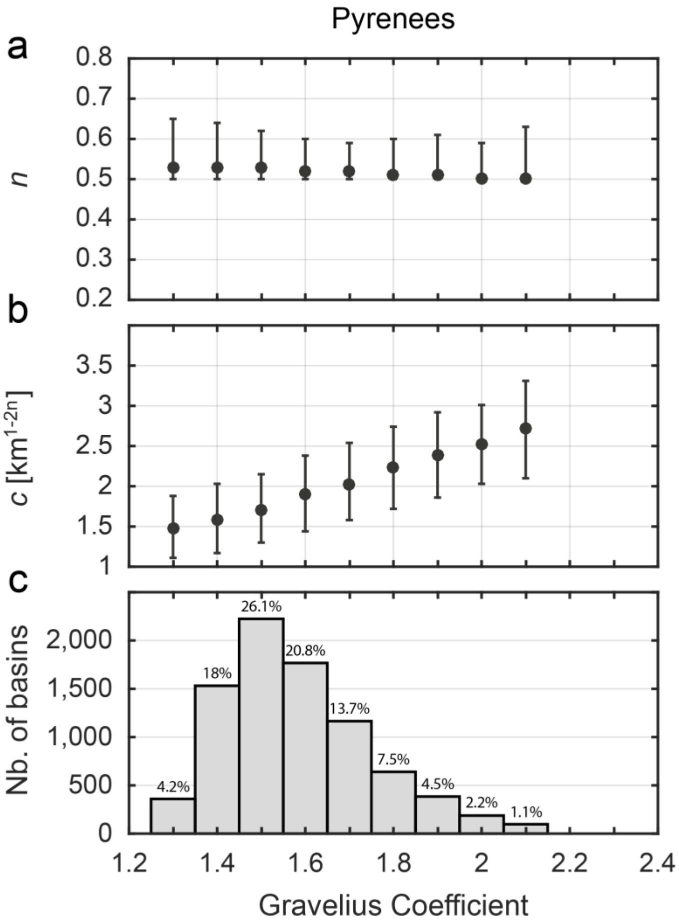

Supplementary Figure 5 | Hack's Law parameters vs. Gravelius coefficient in Pyrenees. Same as supplementary Figure 2, for the Pyrenees.

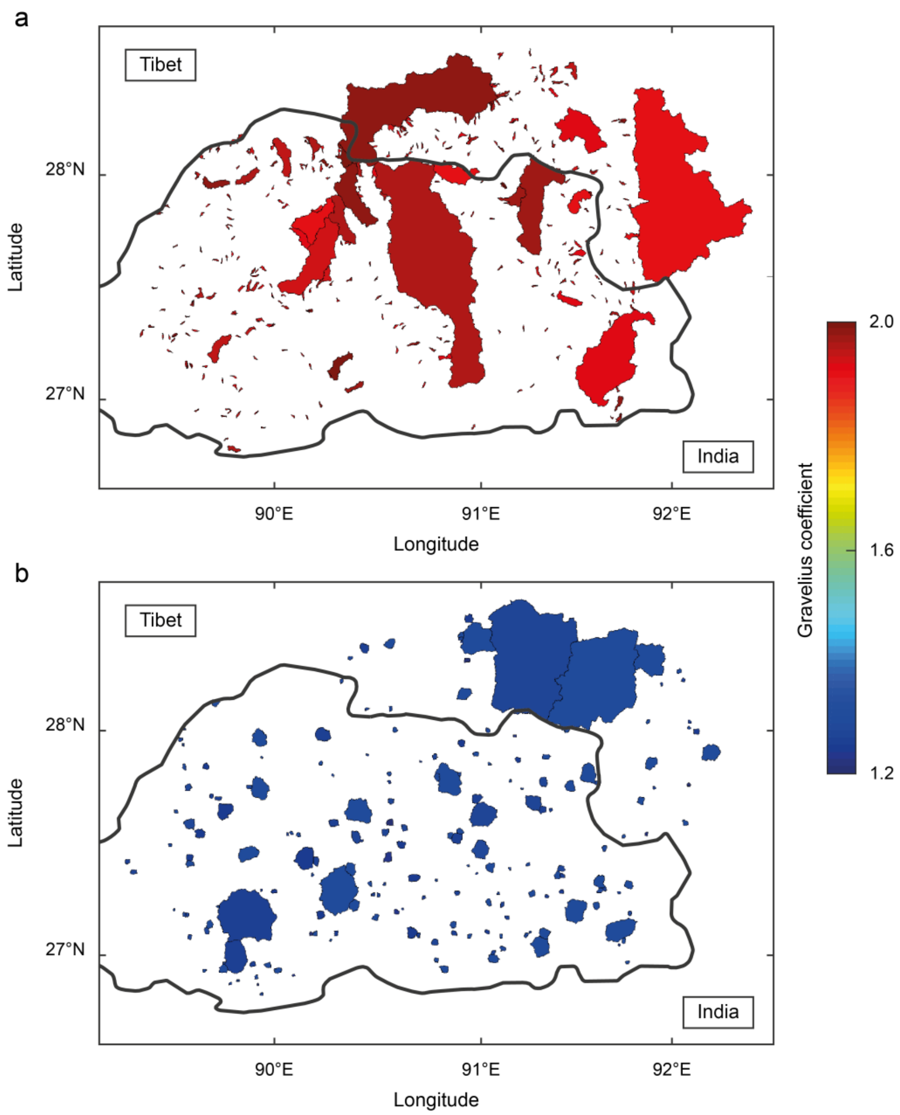

Supplementary Figure 6 | Spatial distribution of drainage basins in Bhutan with respect to their shapes. a, Location of elongated basins with GC ranging between 1.9 and 2.0. **b,** Location of compact basins with GC between 1.2 and 1.3.

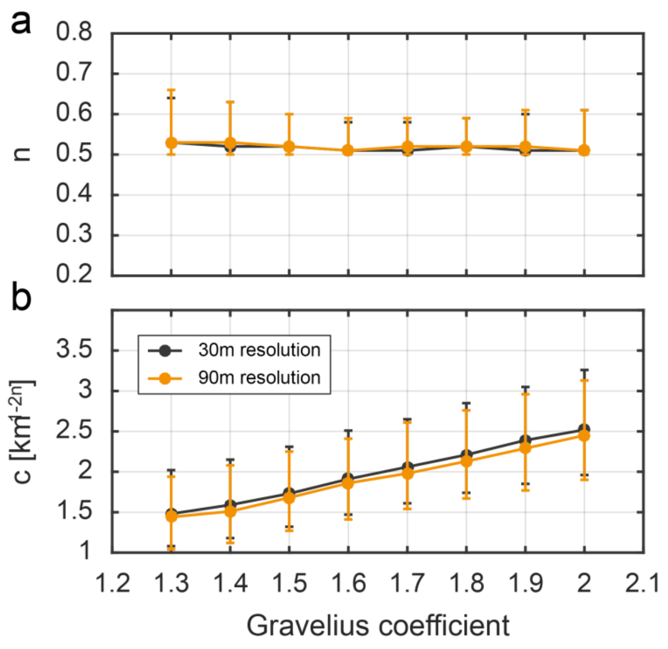

Supplementary Figure 7 | Effect of DEM resolution on Hack's parameters. a and b, Hack's exponent and coefficient obtained in Bhutan Himalaya for 30 m resolution DEM (grey) and 90 m resolution DEM (orange).
